# Supplementary material for: An adversarial collaboration protocol for testing contrasting predictions of global neuronal workspace and integrated information theory
Source: PLoS One. 2023 Feb 10;18(2):e0268577. doi: 10.1371/journal.pone.0268577 (PMC9916582; doi:10.1371/journal.pone.0268577)
Supplement: Attachment — Submitted filename: PLoS ONE Reviewer Comments - Cogitate 2022.docx [file pone.0268577.s001.docx]

Editor & Reviewer comments in **black**; our responses in **magenta**

**Editor’s summary:**

Both the reviewers and I feel that this project is of high value. We are asking for only a minor revision of your manuscript, in keeping with the requirement of PLOS ONE for methodological rigour and completeness. Reviewer 2 has a number of suggestions about how to make the project even better, and I would like to see many, if not most, of these incorporated into your revision (and perhaps even into your preregistered analysis plan). I believe phase 2 of your analysis allows you to make revisions in your process before undertaking phase 3, so this should be possible. I particularly hope that you will seriously consider doing the Bayesian analysis suggested both by reviewer 2 and by me in my additional comments.

We appreciate this guidance and have incorporated almost all of Reviewer 2’s suggestions into our revised manuscript. We will also include these revisions in our next preregistration update which is planned for Aug 2022, prior to entering into phase 3 of our project. In particular, after consulting with experts in Reviewer 2’s laboratory, we now include plans to use Bayesian analyses to evaluate the strength of evidence for and against each theory in our revision. We are very much indebted to the reviewer for this excellent suggestion!

**General instructions for revision:**

Please submit your revised manuscript by Mar 03 2022 11:59PM

Please include the following items when submitting your revised manuscript:

- A rebuttal letter that responds to each point raised by the academic editor and reviewer(s). You should upload this letter as a separate file labeled 'Response to Reviewers'.
- A marked-up copy of your manuscript that highlights changes made to the original version. You should upload this as a separate file labeled 'Revised Manuscript with Track Changes'.
- An unmarked version of your revised paper without tracked changes. You should upload this as a separate file labeled 'Manuscript'.

**Change needed for acknowledgement of funding:**
2. Thank you for stating the following in the Acknowledgments Section of your manuscript:

“This work could not have been possible without the continuous and generous support of the Templeton World Charity Foundation at the different stages of this project to Christof Koch, Michael Pitts, Liad Mudrik and Lucia Melloni, as well as of the Max Planck Society (Lucia Melloni). This project is made possible through the support of a grant from Templeton World Charity Foundation, Inc (TWCF0389) to Lucia Melloni and (TWCF0378) to Liad Mudrik and (TWCF0345) to Michael Pitts. The opinions expressed in this publication are those of the author(s) and do not necessarily reflect the views of Templeton World Charity Foundation, Inc.”

We note that you have provided additional information within the Acknowledgements Section that is not currently declared in your Funding Statement. Please note that funding information should not appear in the Acknowledgments section or other areas of your manuscript. We will only publish funding information present in the Funding Statement section of the online submission form.

Please remove any funding-related text from the manuscript and let us know how you would like to update your Funding Statement. Currently, your Funding Statement reads as follows:

“The authors have declared that no competing interests exist.”

Please include your amended statements within your cover letter; we will change the online submission form on your behalf.

We have included amended acknowledgement and funding statements in our cover letter.

**Editor Comments:**

1. This paper should stand on its own without the need to refer to the preregistered methods and analyses, at least so far as a first reading for the overall sense of the project and the sufficiency of the predictions and data analyses are concerned. My extra (to the reviewers) comments mostly relate to this requirement. Moreover, you should write a sentence or two earlier on stating that readers who wish to view specific analysis plans should see the prereg methods and giving the link there as well as when you do that later in the paper.

We appreciate this suggestion and have revised the Protocol manuscript to better stand on its own while providing the link to the preregistration in the third paragraph of the introduction. We have done so, while still keeping some of the more specific details (e.g., variation of trial numbers per technique) in the preregistration. Thus, the manuscript now stands on its own, and refers to the preregistration where necessary.

2. Re the theories: please give a more extended description of the theories - at least another paragraph or two for each, in which critical terms are defined and a few more references are cited to support choosing these two theories to test. For example, the anatomy of the long-range projections critical to GWS could be described, and a definition of the “cause-effect structure” of IIT could be given (both mentioned by reviewer 2).

Excellent suggestion. We have added an additional paragraph for each theory to more fully describe the central tenants and to define key terms. We also added several references upon first mention of these theories in the introduction to support our choice of testing these two theories in particular.

3. This project is ideal for a Bayesian approach (see reviewer 2 comments) in which the various experiments can provide evidence for or against (or neutral) for each of the theories. At the end we should have a posterior probability that each of the theories is a good one for the NCC. This won’t be a final answer, of course, at least partially because other theories predict some of the same outcomes. But at least there should be a sense of which of the two tested here, if either, is more promising. Mixed results, of course, might favour neither theory, and this too would be a useful outcome, with final odds near 1:1. I strongly encourage you to at propose (and perform) a complementary analysis of the results based on an iterative Bayesian approach. You can begin with a neutral prior and see where the data take you.

Thank you (and reviewer 2) for this suggestion. Indeed, we now plan to use Bayesian analyses to assess the posterior probability of each theory given the evidence. We describe our approach in response to reviewer #2’s comments below, and have amended the Protocol manuscript accordingly.

4. Re multivariate decoding in Experiment 1: please mention here which decoding scheme will be used, especially for M-EEG (linear support vector machine, as mentioned in preregistered methods- could consider using a Bayesian SVM?).

Our plan is to use the first half of the data to optimize our analyses, including which particular decoding scheme we will use, and then lock these analysis details in place, update and publicly share the pre-registration, and only then analyze the second half of the data. SVM (and Bayesian SVMs) are indeed possible (and likely) options, but we also plan to evaluate others (e.g., LDA, naive Bayes, K-nearest neighbor, logistic regression, random forest). We have revised the Protocol manuscript to make this strategy clear, and now list the decoding schemes we plan to consider during the analysis-development stage.

5. Also, please describe how cortical sources will be localized (i.e., if and how you will move from sensor space to source space, via independent component analysis, beamforming, …)?

This detail has been added to the revised Protocol, which now reads as follows:

*“Source localization.* Source localization will be performed using beamforming (LCMV or DICS) or minimum-norm estimation approach (MNE, dSPM). Source-level analyses will be performed in each subject, co-registered to individual anatomical MRIs using the digitized head surface and anatomical landmarks. Time-resolved multivariate decoding will be performed in the sensor space, from which the decoding weights are obtained for each sensor. Source localization will then be performed on the weight maps to project the decoding results to source space (Haufe et al., 2014). To verify the cortical source localization for the decoding results in M-EEG, we will examine whether face/object decoding is localized to the corresponding selective areas, e.g., FFA, PPA. In addition, we will consider using fMRI’s decoding results to localize ROIs for M-EEG decoding analysis.”

6. Also, how will inter-subject alignment be accomplished for group analyses? Please state that here and provide a citation.

This has also been added to the revised Protocol, which now reads as follows:

*“Inter-subject alignment.* Individual data will first be aligned to a standardized brain template (i.e. the ‘fsaverage’ template) before the group analysis. We plan to use two inter-subject alignment methods and test which one is better during phase 2 (analysis development). One approach is to generate the forward (the leadfield) with the spacing between the sources constrained to the MNI “fsaverage” brain (i.e., the same number of sources per area independently of the individual differences). The second approach is to create the leadfield from the individual brain using an even spacing (i.e. the number of sources will depend on the size of each specific areas). Both approaches will be explored and compared during the analysis development stage.”

7. Moreover, decoding M-EEG, especially re cortical sources, is not nearly as advanced as that of fMRI or of iEEG (e.g., work of Knight lab). Results of machine learning even for sensor space can be controversial (i.e., cf. Cruse et al, 2011, The Lancet vs Goldfine, et al, 2013, The Lancet). Yet hypotheses re timing (300-500 msec window) depend on either iEEG or M-EEG. Please state how cortical source decoding of M-EEG data will be verified (via co-registered fMRI?).

Thank you for raising this important concern. We agree that M-EEG decoding, especially considering its cortical sources, is associated with uncertainties due to the inverse problem of source modeling. Meanwhile, as also mentioned by the editor, M-EEG decoding would constitute temporal information that is crucial to test the hypotheses. To verify the cortical source localization for the decoding results in M-EEG, we will examine whether face/object decoding is localized to the corresponding selective areas, e.g., FFA, PPA. Alternatively, as suggested by the reviewer, we will also consider using fMRI’s decoding results to localize ROIs for M-EEG decoding analysis. These strategies have been added to the revised manuscript.

8. Expt 1, analysis 3: You propose to determine interareal information sharing using the weighted phase lag index (general methods). Will this be between sources? How will these be determined from M-EEG data? Why not also use Granger causality or transfer entropy to obtain directed information flow?

Thank you for raising this question. We will update the preregistration with the details of the connectivity analyses in the next major update between phase 2 and 3 of the project (anticipated release in Aug 2022). Regarding the specific question, we will use pairwise phase consistency (PPC) and it will be computed between ROIs. Our current plan is to determine the ROIs sources in the following way:

Some regions of interest (ROIs) - V1, V2, inferior frontal gyrus (IFG), and middle frontal gyrus (MFG) - will be identified using the Destrieux atlas (Destrieux et al., 2010). A functional localization procedure will be used to localize fusiform face area (FFA) and lateral occipital complex (LOC) based on the difference between the face and the object condition when considering the gamma-band activity. For each ROI, source-space time courses will be extracted by averaging all the vectors in a given ROI at each time point to the dominant orientation. If a specific vertex orientation is more than 180° different from the dominant one, sign-flip will be applied.

As for using effective connectivity metrics (e.g. Granger causality), the hypotheses have not been developed to make predictions on directionality.

9. Re MEG head position: MEG is very sensitive to head position and head position can vary considerably during runs. Measuring at the beginning and end of runs is the old way to control for head position, with reruns being necessary when head position varied by more than a small amount. Modern machines usually can measure head position much more often and record it so that compensation can be made computationally and all data retained. Why is this not being done here?

The reason for not using continuous head position measurement is that it can introduce noise in the data. The MEGIN system measures the head position using 4-5 coils placed on the participant's head. During the measurement, high frequency (>200 Hz) signals are produced by those coils and the localization of these signals is used to estimate the head position in the sensor space. However, the interaction between the signals generated by these coils can be non-linear and produce some artifacts that are difficult to filter out. To avoid this issue, we decided to do the head position measurement only during resting periods.

**Reviewer #1 Comments:**

This is interesting, stimulating and very important paper which aims to advance our knowledge on the neural correlates of consciousness (NCC). Specifically, it outlines a collaboration protocol for testing contrasting predictions of global neuronal workspace (GNW) and integrated information theory (IIT) in relation to phenomenon of consciousness.

The project will focus on conscious vision in human subjects and involve two experiments, using three complementary methods: functional magnetic resonance imaging (fMRI), simultaneous magnetoencephalography & electroencephalography (M-EEG) and intracranial electroencephalography (iEEG). All relevant hypotheses, predictions, and planned methods are already preregistered and published at OSF: <https://osf.io/mbcfy/>

To my opinion and considering the format of the paper – Protocol – the paper is ready for publication.

We appreciate Reviewer #1’s positive assessment of our project. We hope that with the revisions based on the editor’s and reviewer #2’s comments that, indeed, this protocol is now ready for publication.

**Reviewer #2 Comments:**

I enjoyed reading this comprehensive and accessible description of an adversarial collaboration to test GNW against IIT predictions of the neuronal contents of consciousness. This is an intriguing endeavour and there has clearly been an enormous amount of work behind-the-scenes, in putting this international and interdisciplinary project together. I thought that you did a great job in summarising the issues you have been contending with.

I am not sure whether it is appropriate to critique the conclusions of your discussions at this stage; however, I have some comments about your data analysis – which I will review from a practical perspective. Otherwise, I have a few minor points that might help clarify the presentation of your proposal for the general reader.

Major points

Your motivation, experimental design and protocols are all very impressive. However, this is not reflected in the quality of your proposed data analysis. Most of your analytic approaches may not be fit for purpose to fulfil the objective of the adversarial collaboration. The current data analysis streams can be critiqued at two levels:

1. First, in testing the predictions of two or more hypotheses, you will need to present the evidence for both hypotheses, in each dataset. The evidence is just the likelihood of the data, under GNW or IIT predictions. This means you need to present Bayes factors (as opposed to classical statistics). Bayes factors will then allow you to assess the evidence for each hypothesis and, crucially, be able to say when there is no evidence for one over the other, given the data at hand (if the Bayes factors are suitably small). This is a key point that contrasts with classical inference, where one can never accept the null hypothesis of no difference. You may want to discuss issue with people who are experienced in Bayesian analysis of fMRI and (i)EEG data – and identify a coherent analysis stream that allows you to compute the requisite marginal likelihood or model evidence, under your carefully identified hypotheses.

This is an excellent suggestion, and after discussion with several experts in Bayesian analyses of fMRI & M-EEG data (including researchers from Reviewer #2’s laboratory), we have decided to arrange a consultant who will work with the teams to instantiate those analyses while keeping time and methods development constraints in mind. During the current phase of the project (i.e., phase 2 analysis optimization), the analysis plans as well as the statistical procedures are being finalized. The result of that optimization phase will be the amendment of the preregistration; then, the second half of the data will be analyzed based on the preregistered analysis plan. As we explain above, we plan for this amendment to include a detailed description of the Bayesian analysis. Notably, implementing such an approach on our project is not an easy feat; besides integrating across multiple labs, multiple modalities and multiple predictions, we should also quantify the relative importance of each prediction per modality, and build a model that takes all these contingencies into account. As this process involves both conceptual work with the adversaries and analysis development with the teams, it requires time to establish and finalize. Yet it is our explicit goal to complete the development of this analysis by the amendment of the preregistration, when all changes will be introduced and consolidated.

2. The second level at which your data analysis falls short is the use of decoding and RSA. Implicit in the use of decoding are constraints that could make your job very difficult. One obvious constraint is that decoding is generally limited to testing for main effects (i.e., targets versus non-targets, seen versus unseen, et cetera). This is a problem because you need to test for interactions: for example, the interaction between stimulus category and task – or between cortical area and task.

I imagine that you hope to use decoding accuracy (or correlations between vectorised RSA matrices) as summary statistics – and then test for interactions using classical inference (e.g., t-tests). The problem is that these kinds of summary statistics are notoriously inefficient (by the Neyman-Pearson lemma).

Furthermore, some of your hypothesis tests rest upon accepting the null. For example, "GNW predicts that conscious content will be decodable from the global workspace, independently of task”. However, using classical inference, you cannot accept the null hypothesis of no difference between classification accuracy between task-relevant and task-irrelevant conditions.

The only analysis that seemed appropriate was the ANOVA of responses to stimuli reported as seen and unseen and an interaction with time, “with the latter testing a key prediction of GNW”. If you could use this kind of analysis throughout, I think you would be on a much stronger footing.

I make these observations because, I can see – in a year or so's time – a beautifully articulated report that ends up with a succession of: "Unfortunately, we could not reject the null hypothesis…"

I do not know whether it is appropriate to address these issues in the current paper. Perhaps it would be sufficient to add some qualifications to the discussion along the above lines?

Thank you for this important comment. To clarify, for the key decoding analyses in Experiment 1, we plan to test for cross-task generalization of decoding. That is, we will train classifiers on data from the task-irrelevant condition (e.g., faces vs. objects), and test them on the data from the task-relevant condition (e.g., faces vs. objects), and vice versa (train on relevant and test on irrelevant). With this approach, the problem of statistically testing for an interaction (e.g. between category and task) goes away, as both factors are integral to the decoder training/testing scheme from the start.

For Experiment 2, the prediction that states, “GNW predicts that conscious content will be decodable from the global workspace, independently of task”, does not depend on testing the null hypothesis of no difference in classification accuracy between task-relevant and irrelevant conditions. In fact, we fully expect decoding accuracy to be higher (in many brain areas) in the task relevant condition compared to the task irrelevant condition. The key tests of this prediction are decodability *per se* (e.g., above-chance decoding of category) in each task separately, and generalization of decoding across tasks (for all seen trials). Relative differences in decoding accuracy are acceptable and are not the focus of this analysis.

Based on the suggestions above regarding Bayesian inference as an optimal approach for assessing the weight of evidence for each theory, we are now confident that the end result will be more informative and nuanced and will avoid a succession of “failures to reject the null hypothesis”.

Minor points

3. On page 2, you say that neurons in prefrontal and cingulate cortices are connected through long-range excitatory axons to high-level sensory areas. While this is true, you attribute the sources of these connections to pyramidal cells of layer 2 and 3. This goes against the usual conception of top-down or descending excitatory connections to sensory levels, which are thought to be deep pyramidal cells. Perhaps you could nuance this sentence or provide some references (this issue is relevant because superficial and deep pyramidal cells contribute differentially to some of the signals you will be analysing).

Thank you for this comment. This statement comes directly from Mashour et al. (2020), “At the neuronal level, the GNW hypothesis postulates a key role for large pyramidal cells in cortical layer II/III (Figure 1B) but also a contribution of pyramidal cells in deeper layer V, as illustrated by recent studies (see below).” We have revised the manuscript for accuracy and to clarify where this statement comes from.

4. In the description of integrated information theory, it may be useful to provide some heuristic descriptions of the technical terms – perhaps in a glossary? For example, what is a “cause-effect structure”. More specifically, what is meant by a “structure” in this setting: is it a correlation structure, an adjacency matrix, a conditional probability et cetera? Structures seem to be closely related to the notion of a "substrate". It is the substrate meant to mean neuronal activity or connectivity? Finally, what is meant by "neuronal mechanisms" are these neuronal dynamics, changes in synaptic efficacy or something more abstract? In short, a little primer on the constructs used by IIT would be useful.

Excellent suggestion. In the revised Protocol, we now provide descriptions of technical terms used by IIT.

5. On page 4, could you replace "Our study focuses on the prefrontal cortex (PFC) as part of the NCC" with "Our study focuses on the NCC in the prefrontal cortex (PFC)".

This sentence has been revised as suggested.

6. On page 4, you talk about "activity-silent” states under the GNW. Can I suggest you remove phrase “activity silent”: otherwise, you might confuse people; who will be asking how can you look for the neural correlates of consciousness when they are silent? You could say something like:

"GNW rests upon neurophysiologically plausible belief updating – of the kind associated with predictive coding – with a well-defined dynamics over the conscious experience (e.g., characteristic onset and offset responses). IIT does not share the same commitments and posits that the pattern of neural activity should persist over the duration of a conscious experience."

Good suggestion. We have now clarified our use of the phrase “activity silent”, which means a change in synaptic weights to support memory rather than needing active neurons to retain this information.

7. Please capitalise “covid” in COVID-19.

Done.

8. In describing your experimental designs, it would be helpful to use a more formal description, so that people can get their heads around the factors. For example,:

“Experiment 1 is a nested factorial design with three factors; namely, stimulus category (face, object, letter versus false font), Target category (face versus letter), and duration (500, 1000 one thousand 500 ms). We were particularly interested in interaction between stimulus and target category; i.e., task relevance and the simple main effect of target versus no target in task relevant responses …”

Thank you for this suggestion. We have revised the Protocol accordingly. The adjusted text (at the end of each design section) for each experiment now reads as follows:

“Thus, in formal terms, Experiment 1 is a nested factorial design with four factors: stimulus category (face, object, letter, false-font), task relevance (task-relevant targets, task-relevant non-targets, task-irrelevant stimuli), stimulus duration (500, 1000, 1500 ms), and stimulus orientation (front view, side view). We are particularly interested in the interactions between stimulus category and task-relevance, as well as between stimulus duration and task-relevance.”

“Formally, Experiment 2 uses a nested factorial design with three factors: stimulus category (faces, objects), stimulus location (left, right), and task (distracted attention, attended). An additional factor will be measured but not manipulated: visibility (seen, unseen), which will be based on subjects’ reports in the distracted attention condition. We are particularly interested in the interactions between stimulus category (and location) and visibility, as well as stimulus category (and location) and task.”

9. On page 10, you say that GNW predicts patterns of activity in the first time window to differ from patterns in later time windows (in contrast to IIT). You then say:

"In RSA, high similarity should be found between content specific patterns of activity in the first three time windows independent of any initial transient. This activity should differ from that found during the fourth time window when the stimulus is no longer presented."

I suspect many readers will find this difficult to unpack because you seem to be talking about [dis]similarities of similarities. It would be simpler and more efficient to simply test for interactions between content and time windows using standard multivariate procedures (i.e., canonical variates analysis – CVA). CVA is a general linear model that can be regarded as a multivariate generalisation of an ANOVA. You can then test directly for the different hypotheses about time specific responses in spatially distributed patterns. I mention this because open source CVA code is available (e.g., spm_CVA.m) that returns approximations to log evidence (based on the AIC and BIC).

We agree with the reviewer that the current formulation of the analysis of neural activation associated with sustained percepts is unclear and we have revised it accordingly. We believe that the current analysis plan is very close to the one suggested by the reviewer.

The current plan when analyzing ECoG data is to divide this into two independent analyses, one geared to investigate the temporal profile of the neural responses to stimuli of different durations, and a second one addressing the informational content of the neural responses as a function of time. For the first analysis, the predictions of the theories will be tested using a linear mixed model framework. Activation on a single trial basis will be aggregated in three time windows (0.8-1.0s, 1.3-1.5s and 1.8-2.0s) both in the high-gamma and the ERP signals. Theories’ predictions will be translated into two separate models, which will be fitted to the observed data to investigate the explanatory power of each theory. Predictors with values of 1 indicate activation higher than baseline, and predictors with values of 0 indicate activation at baseline. The patterns of those binary predictors will reflect the neural activations predicted by each of the theories for the duration of each stimulus. The null model will take stimulus duration (0.5, 1.0 and 1.5 seconds) and time window (0.8-1.0, 1.3-1.5 and 1.8-2.0 seconds) as fixed effects and trials as a random effect. Two additional models will be fitted, each taking on theory-specific predictors as an additional fixed effect. Finally, two other models will be computed taking each theory’s predictors and category (e.g., face, object) as well as the interaction between theories predictors and category as fixed effects. AIC values will be used to determine the model that best fits the data. We believe this approach is very similar to the CVA analysis suggested by the reviewer.

In a second analysis, we will focus exclusively on trials in which stimuli were presented for a duration of 1.5 seconds. On those trials, we will perform decoding analysis (e.g., faces vs objects), and evaluate the temporal generalization of the decoders. The decoding will be performed over regions of interest defined by the adversaries in the Destrieux Atlas. For each ROI, the resulting temporal generalization matrices will be subsampled in the time windows of interest (0.3-0.5, 0.8-1.0, 1.3-1.5, 1.8-2.0 seconds). Two model matrices, one per theory predictions, will be derived by setting to 1 the time*time coordinates where theories predict significant generalization of decoding and 0 elsewhere. The temporal generalization matrix will then be compared to the two predicted matrices through partial correlation (correlating one predicted matrix while regressing out the other). The resulting partial correlation values between the observed and predicted matrices will then be centered on 0.5 (defined between 0 and 1) and subtracted from one another, such that a positive value indicates better fit of the IIT matrix while a negative value indicates better fit of the GNW matrix. The significance of the difference is then investigated through permutation testing, generating a null distribution of partial correlation differences by shuffling the test set labels 1028 times prior to temporal generalization computations. In case of significantly different values, the sign is investigated to assess which of the two theories predicted such differences. This method is complementary to the linear mixed model approach described above.

The revised protocol manuscript now reads as follows:

“Here, we will evaluate the neural responses associated with the maintenance of a percept in consciousness. Specifically, we will investigate the temporal profile of activation evoked by stimuli of different durations as well as the temporal profile of informational content of the neural responses throughout the duration of the stimulus. For the former, we will compare activation levels between conditions (defined by stimulus duration) in four different time windows: (1) 300-500 ms, (2) 800-100 ms, (3) 1300-1500 ms, and (4) 1800-2000 ms using linear mixed models. For the latter, we will combine temporal generalization (35) and representational similarity analysis (RSA; 31) approaches: in long duration trials, stimulus category (as well as orientation) will be decoded in a temporal generalization fashion (testing for generalization of neural patterns containing the content specific information across time). Representational similarity analysis will then be employed to compare the resulting temporal generalization matrix to the predictions of the theories in the specified time windows.

GNW predicts ignitions within PFC following stimulus onset and possibly also during stimulus offset, constituting updates to conscious perception, with virtually no activity between updates (except for occasional stochastic bursts of spontaneous reactivation; 36). Thus, for consciously perceived but task-irrelevant stimuli, neural activation is predicted to reflect an interaction between duration and time window in the PFC, such that activation is maximal in windows following stimulus onset and offset and minimal elsewhere. GNW further predicts that the patterns of activation will be consistent and content-specific between the windows where activation is expected to be maximum, whereas in the windows of minimal activation, there should be no content-specific activation. This translates in expected temporal generalization of decoding accuracy between the onset and offset burst with no generalization in between. This prediction can be translated in a model matrix to be correlated with the obtained temporal generalization results. IIT predicts sustained, content-specific activity patterns in posterior cortex throughout the duration of the percept (after the initial transient response). Therefore, activation levels within posterior areas should be higher for longer-duration (and consciously seen) than shorter-duration (and no longer seen) stimuli during analysis time windows 2 and 3. In addition, temporal generalization of decoding accuracy should be evident for as long as the stimuli are consciously experienced, and will be tested via correlation with a different model matrix than for GNW.”
